# Editorial: Environmental factors, epigenetics, and reproductive health

**DOI:** 10.3389/fendo.2024.1517774

**Published:** 2024-11-19

**Authors:** Wei Wu

**Affiliations:** ^1^ State Key Laboratory of Reproductive Medicine and Offspring Health, Center for Global Health, Nanjing Medical University, Nanjing, China; ^2^ Key Laboratory of Modern Toxicology of Ministry of Education, School of Public Health, Nanjing Medical University, Nanjing, China; ^3^ Taizhou Clinical Medical College, Nanjing Medical University, Taizhou, China

**Keywords:** environmental factors, epigenetics, reproductive health, *in vitro* fertilization (IVF), frozen-thawed embryo transfer (FET), recurrent implantation failure (RIF), infertility, ovarian function and reserve

Embroidered within the rich tapestry of human existence lies the intricate interplay of genetic endowments and environmental pressures, a complex weave that profoundly sculpts the landscape of reproductive health. In this special Research Topic, “Environmental Factors, Epigenetics, and Reproductive Health,” we embark on a quest to decipher the intricate dance between these two forces, exploring how they jointly orchestrate the symphony of reproductive potential and its diverse outcomes.

The environment, in its broadest sense, encompasses a myriad of elements that exert a significant influence on our biological systems. The growing body of evidence linking environmental risk factors to reproductive disorders serves as a clarion call to delve deeper into this field. A spectrum of conditions, from infertility to endometriosis and developmental anomalies, have been correlated with environmental exposures, underscoring the imperative nature of this research (1–3).

Epigenetics, the study of heritable changes in gene expression that do not involve alterations to the DNA sequence, emerges as a central theme within this research endeavor. Epigenetic modifications, such as DNA methylation and histone modifications, are highly responsive to environmental cues and can induce enduring shifts in gene expression. These modifications are particularly pivotal during developmental stages, critically influencing the epigenome—the cellular blueprint of epigenetic marks. Disruptions to this delicate process, often environmental in origin, can lead to aberrant epigenetic patterns, potentially seeding the ground for reproductive and developmental disorders (4, 5).

The developmental origins of health and disease (DOHaD) hypothesis posits that early-life exposures can cast a long shadow over an individual’s health trajectory, including their reproductive future. This concept resonates deeply within the context of environmental impacts on the epigenome. The embryonic and fetal development period is a sensitive window when the genome is exceptionally vulnerable to environmental influences. Toxins, pollutants, and even psychological stress can imprint lasting epigenetic marks, predisposing individuals to a myriad of reproductive health challenges later in life (6).

This Research Topic is a compilation of five original articles, each investigating the causal relationships and molecular underpinnings through which environmental risk factors impinge upon reproductive and developmental health. By synthesizing these studies, we aim to offer a comprehensive vista of the current understanding, challenges, and emerging horizons in this field.

The first article in this series, authored by Du et al., investigates the impact of seasonal and temperature variations on *in vitro* fertilization (IVF) outcomes. Their retrospective cohort study, encompassing over 14,000 oocyte retrieval cycles, suggests that neither season nor temperature significantly influences the cumulative live birth rate or the time to live birth. This study sheds valuable light on the role of environmental factors in assisted reproductive technologies, challenging previous assumptions about the criticality of IVF timing.

Moving from external environmental influences to internal biological processes, Holzer et al. present a case-control study that examines the relationship between iron status and unexplained infertility. Their findings indicate that women with unexplained infertility are more likely to exhibit lower ferritin levels, suggesting a potential link between iron deficiency and fertility challenges. This research paves the way for novel assessments and treatments of infertility, especially in cases defying conventional explanations.

The third article, contributed by Yan et al., explores the use of hyaluronic acid-enriched transfer medium in frozen-thawed embryo transfer (FET) outcomes for patients with recurrent implantation failure (RIF). Their retrospective study proposes that the inclusion of hyaluronic acid in the transfer medium can bolster clinical pregnancy rates among RIF patients. This finding is significant, offering a potential strategy to augment the success rates of FET in a particularly challenging patient demographic.

The fourth article, authored by Xie et al., employs a Mendelian randomization approach to assess the causal association between pregnancy complications and the risk of diabetes and cardiovascular disease. Their study provides genetic evidence that gestational diabetes, pregnancy with abortive outcomes, and hypertensive disorders in pregnancy may serve as early indicators for metabolic and cardiovascular risks. This research is particularly pertinent, given the escalating awareness of the long-term health implications of pregnancy complications.

Lastly, Jiang et al. delve into the effects of chronic stress on ovarian function and reserve. Using a chronic stress model in mice, they reveal that stress can lead to meiotic arrest failure and a decline in ovarian reserve. This study is groundbreaking, offering a mechanistic understanding of how psychological stress can impact reproductive health at the cellular level.

In conclusion, this Research Topic assembles a diverse array of research that underscores the multifaceted nature of reproductive health. From the external influences of season and temperature to the internal dynamics of iron metabolism, from the physical challenges of embryo transfer to the genetic predispositions unveiled through Mendelian randomization, and from the chronic stress that can disrupt ovarian function, each study contributes a piece to the complex puzzle of reproductive biology. The collective findings of Du et al., Holzer et al., Yan et al., Xie et al., and Jiang et al. not only enrich our scientific understanding but also hold the potential to inform clinical practice, steering the development of more effective strategies for supporting reproductive health.

As we gaze into the future, the studies featured in this Research Topic underscore the necessity for sustained research into the environmental and epigenetic factors that sculpt reproductive outcomes. They remind us of the importance of a holistic approach to reproductive health, one that considers the interplay of lifestyle, environment, genetics, and individual biology. It is my hope that this Research Topic will inspire further inquiry and innovation in the field, leading to improved diagnostics, treatments, and ultimately, better reproductive health outcomes for all.

The journey from research to application is often a long one, filled with challenges and uncertainties. However, the potential benefits of this research are immense, offering hope for millions of individuals who grapple with reproductive health issues. As we stand on the brink of new discoveries, it is our collective responsibility as researchers, clinicians, and policymakers to collaborate in harnessing the power of this knowledge to enhance the lives of those affected by reproductive disorders.

In the end, the “Environmental Factors, Epigenetics, and Reproductive Health” Research Topic is more than an academic endeavor; it is a call to action. It serves as a reminder that our environment, in all its complexity, plays an indispensable role in our health and well-being. As we continue to unravel the enigmas of the epigenome and its susceptibility to environmental influences, we inch closer to a future where reproductive health is not a privilege but a right for all.
